# Selenium Toxicity to Honey Bee (*Apis mellifera* L.) Pollinators: Effects on Behaviors and Survival

**DOI:** 10.1371/journal.pone.0034137

**Published:** 2012-04-13

**Authors:** Kristen R. Hladun, Brian H. Smith, Julie A. Mustard, Ray R. Morton, John T. Trumble

**Affiliations:** 1 Department of Entomology, University of California Riverside, Riverside, California, United States of America; 2 School of Life Sciences, Arizona State University, Tempe, Arizona, United States of America; Monash University, Australia

## Abstract

We know very little about how soil-borne pollutants such as selenium (Se) can impact pollinators, even though Se has contaminated soils and plants in areas where insect pollination can be critical to the functioning of both agricultural and natural ecosystems. Se can be biotransferred throughout the food web, but few studies have examined its effects on the insects that feed on Se-accumulating plants, particularly pollinators. In laboratory bioassays, we used proboscis extension reflex (PER) and taste perception to determine if the presence of Se affected the gustatory response of honey bee (*Apis mellifera* L., Hymenoptera: Apidae) foragers. Antennae and proboscises were stimulated with both organic (selenomethionine) and inorganic (selenate) forms of Se that commonly occur in Se-accumulating plants. Methionine was also tested. Each compound was dissolved in 1 M sucrose at 5 concentrations, with sucrose alone as a control. Antennal stimulation with selenomethionine and methionine reduced PER at higher concentrations. Selenate did not reduce gustatory behaviors. Two hours after being fed the treatments, bees were tested for sucrose response threshold. Bees fed selenate responded less to sucrose stimulation. Mortality was higher in bees chronically dosed with selenate compared with a single dose. Selenomethionine did not increase mortality except at the highest concentration. Methionine did not significantly impact survival. Our study has shown that bees fed selenate were less responsive to sucrose, which may lead to a reduction in incoming floral resources needed to support coworkers and larvae in the field. If honey bees forage on nectar containing Se (particularly selenate), reductions in population numbers may occur due to direct toxicity. Given that honey bees are willing to consume food resources containing Se and may not avoid Se compounds in the plant tissues on which they are foraging, they may suffer similar adverse effects as seen in other insect guilds.

## Introduction

Over 60% of the world's crop species are animal pollinated, with honey bees constituting a large component [1], [2]. The value of the honey bee (*Apis mellifera* L., Hymenoptera: Apidae) as managed pollination services in the United States is estimated to be up to 14 billion dollars per year [3]–[5]. Declines in honey bee populations due to pesticide poisoning have been a focus of recent research [6], but the role of soil-borne pollutants on honey bee survival has not been examined. Few studies have focused on the toxicological effects of metal or metalloid pollutants on bee behaviors and survival.

Honey bees forage over very large areas and bring plant materials (nectar, pollen and propolis) back to their hives, and thus may collect significant amounts of toxic contaminants. Plant pollinators such as honey bees and their honey products have been investigated as potential bioindicators of metal and metalloid pollutants [7], [8]. Varying amounts of contaminants that are toxic to insects have been found in honey, propolis, and pollen from honey bee hives located in close proximity to polluted sites around the world [9]–[15]. With regards to the soil-borne pollutant, selenium (Se), pollen collected by bees from plants growing in fly ash from coal-burning electrical power plants contained 14 mg Se kg^−1^ [16]. In an urban, uncontaminated area of Poland, honey bee foragers collected from stationary hives contained 7.03 mg Se kg^−1^ [17]. Honey collected from different regions of Turkey contained 38 to 113 µg kg^−1^ [18]. Honey collected from hives located in seleniferous areas of Colorado contained up to 0.73 mg Se kg^−1^ [19]. These findings raise the following issues: 1) Do nectar and pollen from plants growing in high metal or metalloid soils contain levels of these elements that, when collected, are toxic to brood or workers? 2) What is the potential for adverse effects on pollinator health of widespread contamination of selenium? Although there has been some interest in using honey bees and their products as bioindicators of pollution, few studies have examined the effects of foraged plant tissues containing soil-borne pollutants such as Se on pollinator health.

Selenium (Se) is a metalloid that occurs naturally in certain alkaline soils from shale deposits of prehistoric inland seas [20]. Agricultural water drainage dissolves Se from these naturally seleniferous soils and has caused the buildup of selenate (SeO_4_^2−^), the predominant and bioavailable form of Se. One of the worst cases of Se pollution occurred at the Kesterson Reservoir in the San Joaquin Valley (Merced County, California, USA), a major drainage site for many agricultural regions of California [21]. The EPA maximum contaminant level (MCL) of 0.05 mg L^−1^ for Se in drinking water was based on evidence from this well-documented case of Se poisoning as well as 96 hour acute and chronic toxicity testing of aquatic animals. However, the MCL does not consider bioaccumulation or biomagnification of Se. Studies have demonstrated the biomagnification of Se throughout the food web [22], but few studies have examined the effects of plants and the insects that feed on them in Se-contaminated sites. However, in several studies examining Se levels in arthropods collected from accumulating plants, various floral visitors contained up to 75 µg Se g^−1^ dry weight (dw) [23], honey bees contained 14.8 µg Se g^−1^ dw and bumble bees contained 251 µg Se g^−1^ dw [19]. Thus, there is the possibility for biotransfer of Se from plant to pollinator.

Despite its toxic properties, selenium is also a micronutrient that is essential to many organisms, including mammals, fish, and bacteria [24], but slightly higher levels can cause toxic effects. Selenium's toxicity is attributed to its similarity to sulfur. Se replaces sulfur in amino acids such as cysteine and methionine and can change protein folding, disrupt cell metabolism [25], [26], and alter the activity of enzymes if the Se replaces S near the active site [27]. Inorganic forms of Se can also cause oxidative stress [28] and DNA damage [29]. Although Se is a micronutrient for many living organisms, a surplus of the element can cause developmental deformities and toxicity.

There is good evidence that Se accumulation can have negative effects on plant growth, insect herbivores, their predators and parasites, and the detritivores that feed on decaying plant and animal tissues [30], [31], yet we know very little about how pollutants such as Se impact pollinators. Herbivores fed plant tissues containing high levels of metals, metalloids (such as Se), or other accumulated elements have shown reduced development and survival [32], and several studies have shown some insect species can not detect detrimental levels of Se [33], [34], but there are no studies to date examining the effects of Se-containing floral tissues on insect pollinator behaviors and survival.

Our overall objective was to determine whether the two main forms of Se commonly found in accumulating plants, selenate and selenomethionine [35]–[37], can have sublethal or lethal effects on the honey bee (*Apis mellifera* L., Hymenoptera: Apidae). Our first objective examined whether the presence of Se affected honey bee gustatory behavior via two different chemosensory organs (antenna or proboscis). Our second objective was to examine whether Se has sublethal effects on the honey bee's feeding behaviors, particularly if it can alter the bee's responsiveness to sucrose. Our third objective tested whether increasing concentrations of Se can cause mortality when administered as a single or chronic dose to honey bee foragers. If pollinators cannot detect and avoid Se compounds in the pollen and nectar on which they are foraging and collecting for their progeny, they may suffer similar adverse effects as seen in other insect guilds.

## Results

### Antennal response assays

**Figure 1:**
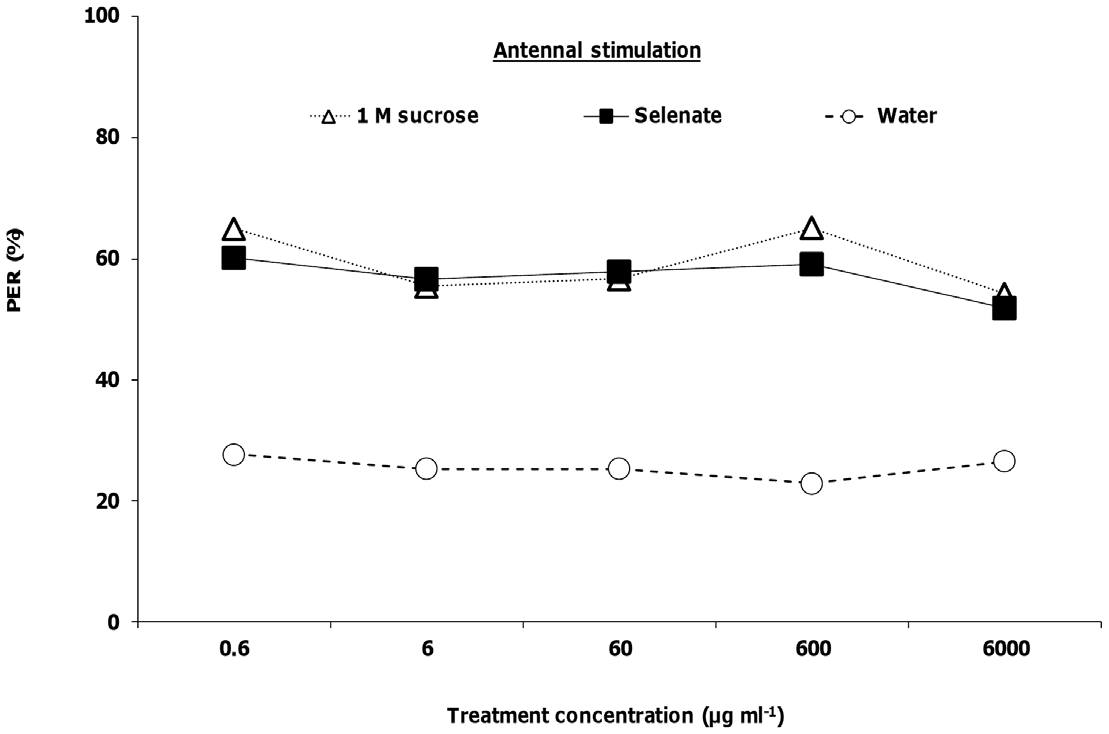
Honey bee behavioral responses to antennal stimulation with selenate. Honey bees were stimulated with 1 M sucrose, water, and selenate in 1 M sucrose (N = 83). Asterisks indicate significance of *P<0.05, **P<0.001, ***P<0.0001 (Logistic regression with multiple comparisons) between 1 M sucrose and treatment lines.

**Figure 2:**
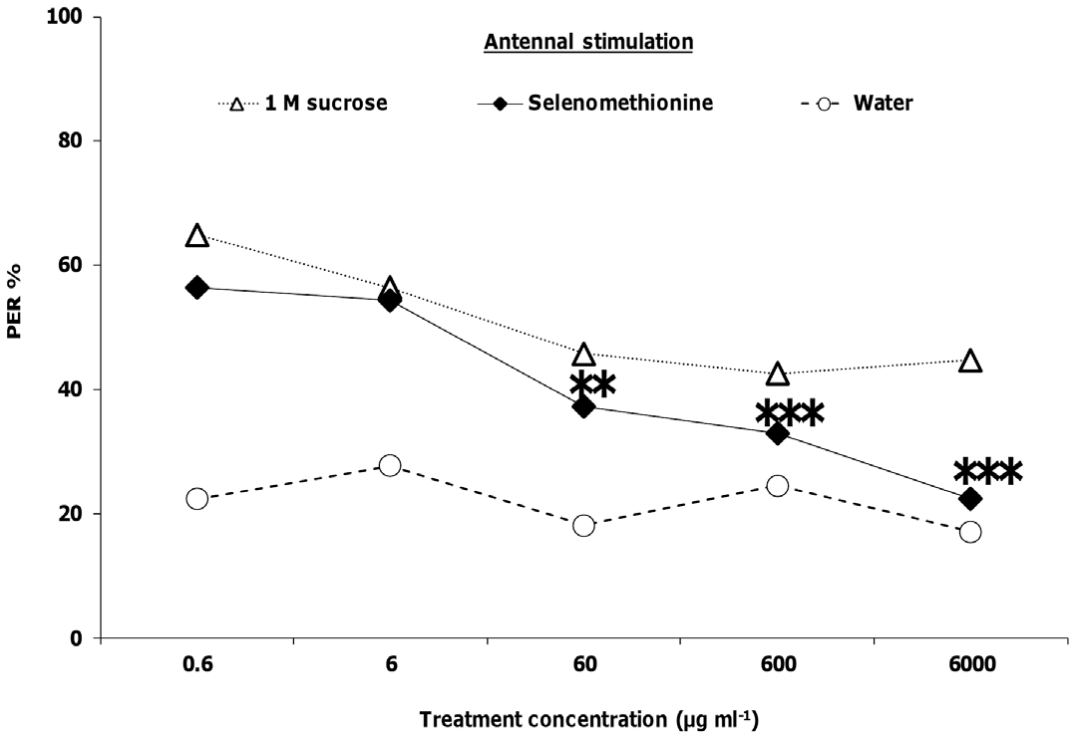
Honey bee behavioral responses to antennal stimulation with selenomethionine. Honey bees were stimulated with 1 M sucrose, water, and selenomethionine in 1 M sucrose (N = 94). Asterisks indicate significance of *P<0.05, **P<0.001, ***P<0.0001 (Logistic regression with multiple comparisons) between 1 M sucrose and treatment lines.

**Figure 3:**
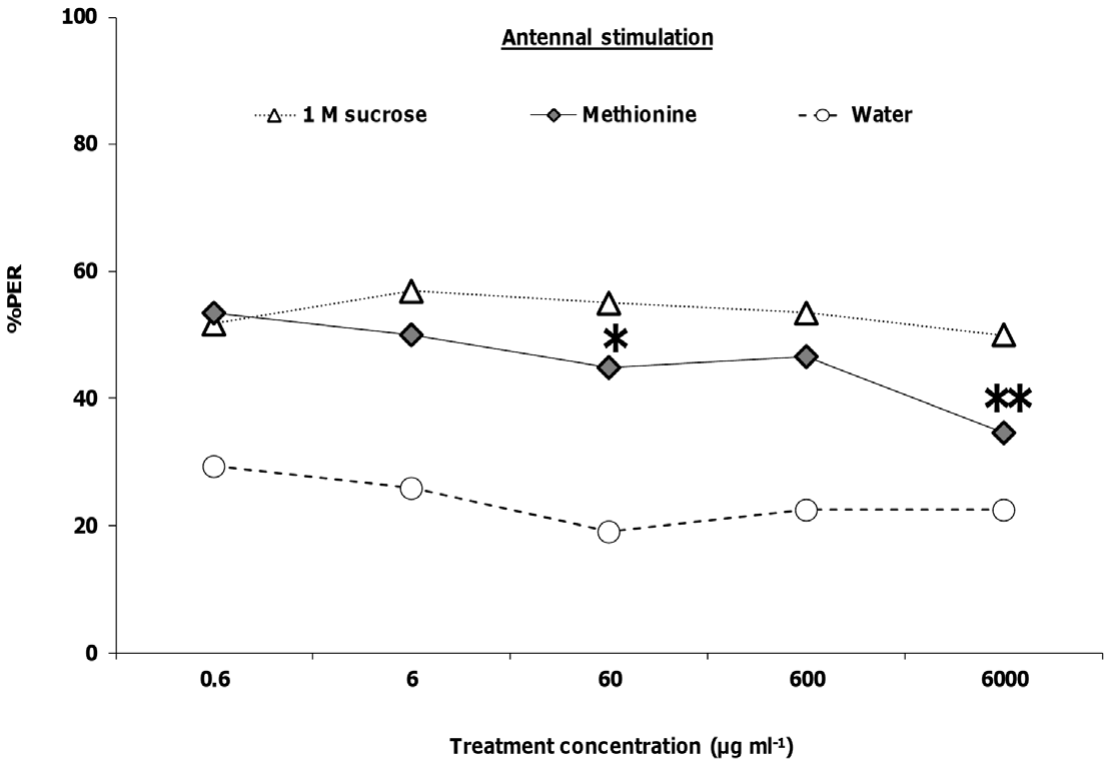
Honey bee behavioral responses to antennal stimulation with methionine. Honey bees were stimulated with 1 M sucrose, water, and methionine in 1 M sucrose (N = 58). Asterisks indicate significance of *P<0.05, **P<0.001, ***P<0.0001 (Logistic regression with multiple comparisons) between 1 M sucrose and treatment lines.

The proboscis extension reflex (PER) involves stimulating a honey bee's antennae with a sucrose solution. The bee will then reflexively extend its proboscis in response to the stimulation. We examined whether honey bees exhibited a reduced PER response to sucrose solutions that contained selenate, selenomethionine or methionine over a range of concentrations spanning five orders of magnitude, from 0.6 to 6000 µg ml^−1^. Honey bee foragers' PER responses to antennal stimulation by selenate were not significantly different than the responses to 1 M sucrose at any of the 5 concentrations (logistic regression, *Χ_6_* ^2^<3.43, *p*>0.06 for all; Figure 1). Responses to selenomethionine plus sucrose were significantly lower than the 1 M sucrose control at 60 µg ml^−1^ (*Χ_1_* ^2^ = 11.80, *p*<0.001), 600 µg ml^−1^ (*Χ_1_* ^2^ = 22.40, *p*<0.0001) and 6000 µg ml^−1^ (*Χ_1_* ^2^ = 46.51, *p*<0.0001; Figure 2). For methionine, responses were only significantly lower than the 1 M sucrose control at the 60 µg ml^−1^ (*Χ_1_* ^2^ = 4.19, *p*<0.05) and 6000 µg ml^−1^ treatments (*Χ_1_* ^2^ = 8.15, *p*<0.001; Figure 3). PER responses to antennal stimulation with solutions containing 1 M sucrose plus selenate (Figure 1) or methionine (Figure 3) were significantly higher than responses to water at all 5 concentrations (*Χ_6_* ^2^>6.75, *p*<0.01 for all). Responses to antennal stimulation by sucrose plus selenomethionine were significantly higher than responses to water at the 4 lowest concentrations (*Χ_5_* ^2^>11.42, *p*<0.001 for all; Figure 2). However, at the 6000 µg ml^−1^ concentration, the response (22%) was not significantly different from that for water (17%, *Χ_1_* ^2^ = 0.02, *p* = 0.88).

### Proboscis response assays

**Figure 4:**
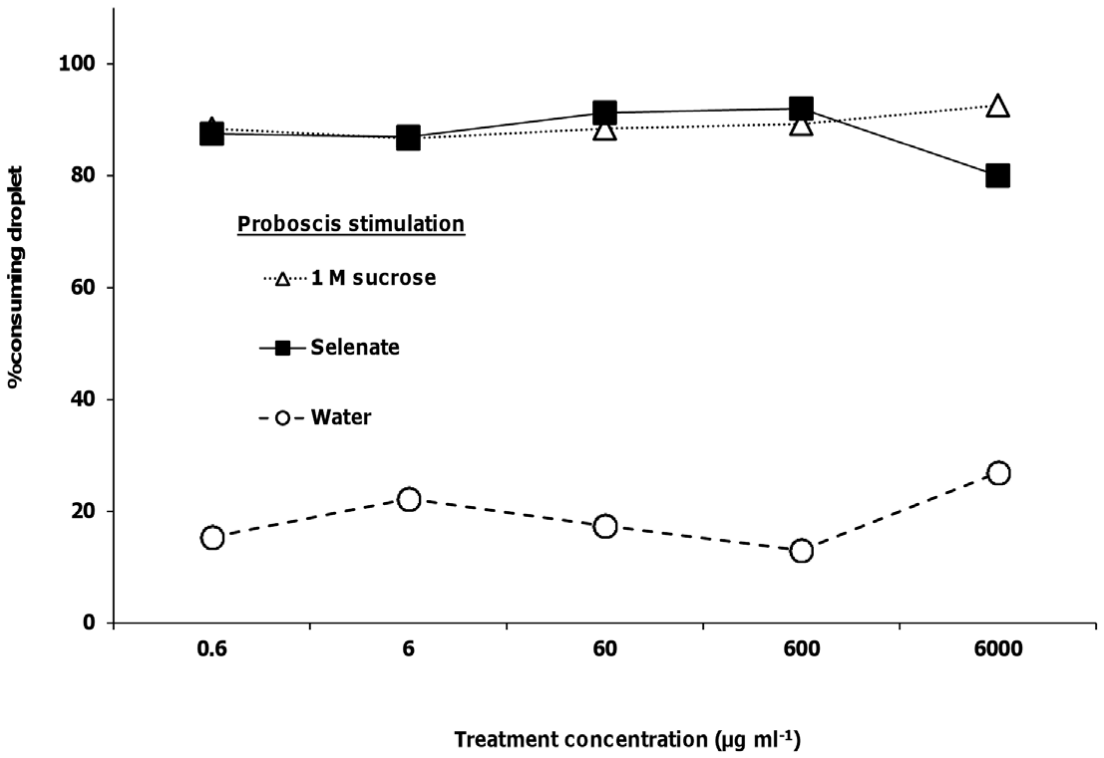
Honey bee behavioral responses to proboscis stimulation with selenate. Honey bees' proboscises were stimulated with 1 M sucrose, water, and selenate in 1 M sucrose (N = 23–30).

**Figure 5:**
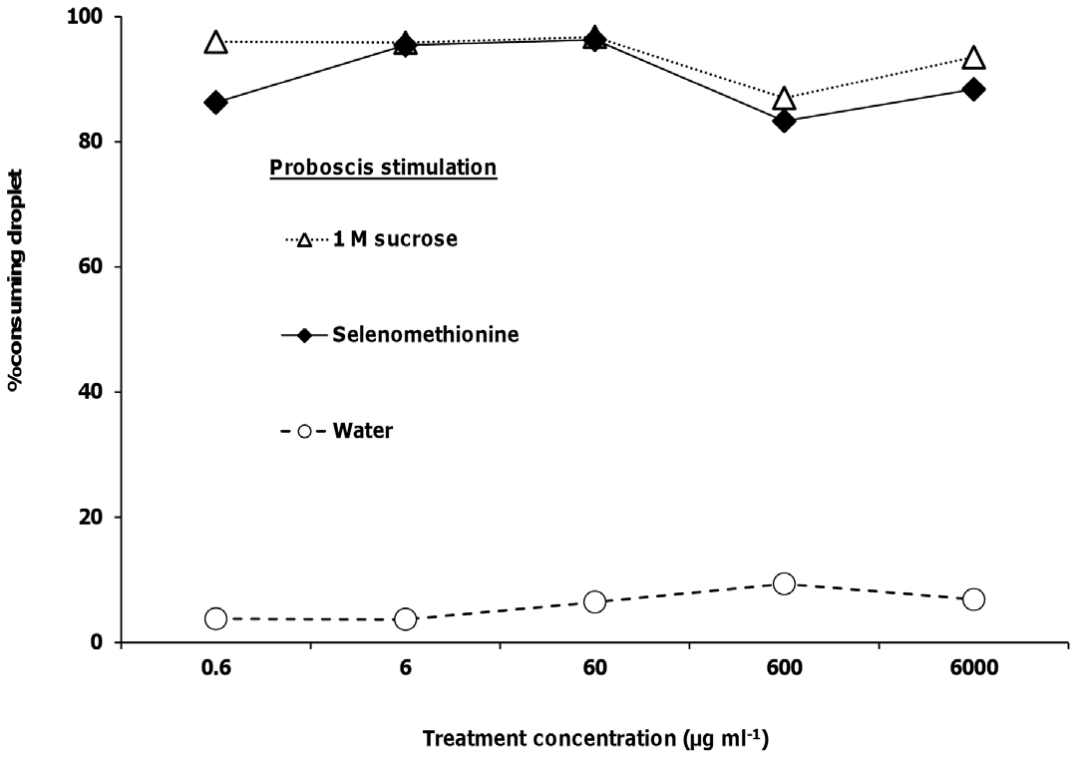
Honey bee behavioral responses to proboscis stimulation with selenomethionine. Honey bees' proboscises were stimulated with 1 M sucrose, water, and selenomethionine in 1 M sucrose (N = 22–31).

**Figure 6:**
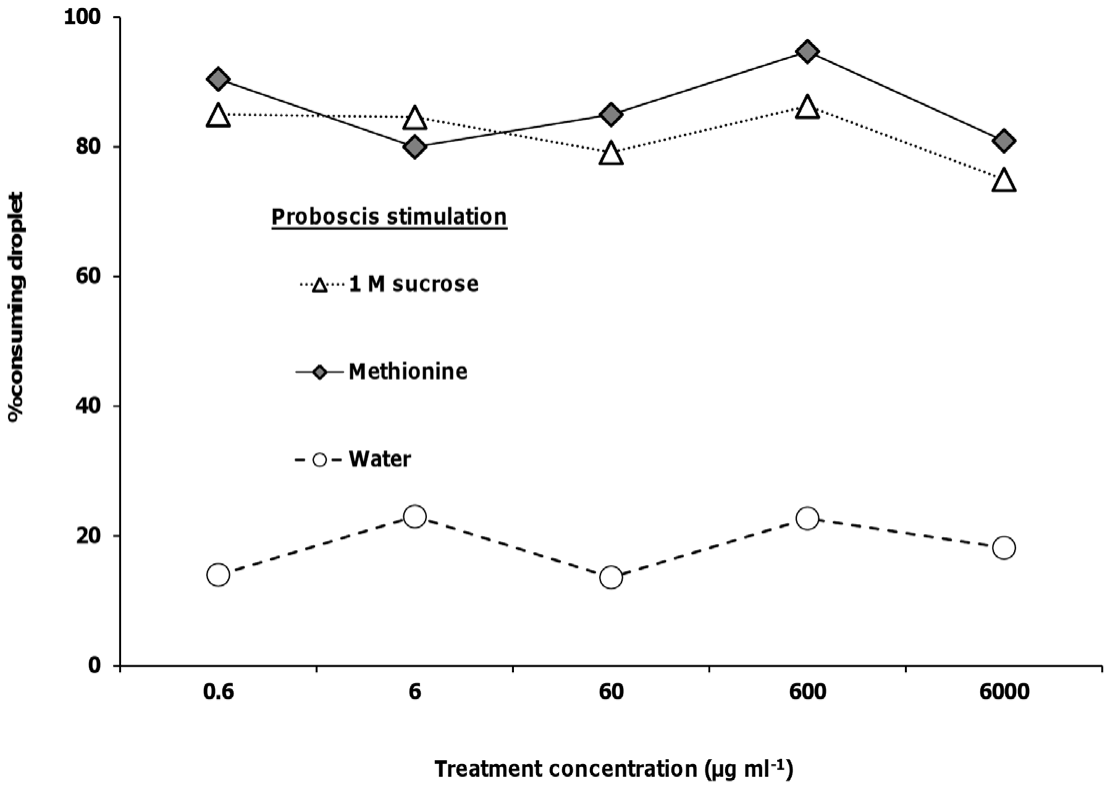
Honey bee behavioral responses to proboscis stimulation with methionine. Honey bees' proboscises were stimulated with 1 M sucrose, water, and methionine in 1 M sucrose (N = 19–26).

As well as receptors on the antennae, honeybees also have gustatory receptors on the proboscis. We examined if the presence of selenate, selenomethionine or methionine, at the same 5 concentrations, affected the willingness of bees to actually consume 1 M sucrose solutions. Bees that were given sucrose solutions containing selenate showed no significant differences in consumption of the droplet between 1 M sucrose and any of the 5 selenate concentrations (logistic regression, *Χ_5_* ^2^<1.45, *p*>0.23 for all; Figure 4). Proboscis stimulation with the water treatment elicited a significantly lower response than 1 M sucrose or any of the 5 selenate concentrations (*Χ_6_* ^2^>13.99, *p*<0.002 for all; Figure 4). There were no significance differences in the percent of bees consuming the droplet between 1 M sucrose and any of the selenomethionine (*Χ_5_* ^2^<0.79, *p*>0.37 for all; Figure 5) or methionine (*Χ_5_* ^2^<0.76, *p*>0.38 for all concentrations; Figure 6) treatments. Consumption responses to proboscis stimulation with water were significantly lower than responses to 1 M sucrose and selenomethionine (*Χ_6_* ^2^>13.99, *p*<0.002 for all concentrations) or methionine (*Χ_6_* ^2^>14.46, *p*<0.0001 for all concentrations).

### Sucrose response threshold assays

The effects of selenate, selenomethionine, and methionine consumption on the responsiveness of honey bee foragers to sugars were determined using sucrose response thresholds (SRT), or the lowest sucrose concentration that elicits a PER response. Bees from all selenate treatment groups showed a dose-dependent change in PER to increasing concentrations of sucrose (logistic regression, *Χ_6_* ^2^ = 58.09, *p*<0.0001, Table S1). The sucrose response threshold occurred between 3 and 10%, except for the group of bees fed 60 µg selenate ml^−1^, whose response to sucrose never significantly differed from that of water. Selenate feeding treatment had a significant effect on proboscis extension response (*Χ_5_* ^2^ = 13.34, *p*<0.02), resulting in a decrease in overall average PER for all selenate feeding treatments (Table S1). The percentage of bees responding with proboscis extension dropped from 48% in bees fed the control (1 M sucrose) to as low as 17% in the 6000 µg ml^−1^ selenate-fed bees. However, there was no significant interaction between the sucrose antennal treatment and the selenate feeding treatment (*Χ_30_* ^2^ = 37.30, *p* = 0.17), indicating that selenate feeding did not alter the sucrose response threshold of 3 to 10% (Table S1).

All selenomethionine treatment groups showed a dose-dependent change in PER to increasing concentrations of sucrose (*Χ_6_* ^2^ = 40.08, *p*<0.0001). The sucrose response threshold occurred between 3 and 10%, except for the 0.6 and 6 µg ml^−1^ treatment groups whose sucrose response thresholds were as high as 30% (Table S1). Selenomethionine feeding treatment did not have a significant effect on sucrose response threshold (*Χ_5_* ^2^ = 4.41, *p* = 0.49). In addition, the interaction of selenomethionine feeding treatment and the sucrose antennal treatment was not significant (*Χ_30_* ^2^ = 41.15, *p* = 0.09).

All methionine treatment groups showed a dose-dependent change in PER to increasing concentrations of sucrose (*Χ_6_* ^2^ = 57.93, *p*<0.0001). The sucrose response threshold occurred between 1 and 10% (Table S1). Methionine feeding treatment did not have a significant effect on sucrose response threshold (*Χ_5_* ^2^ = 7.98, *p* = 0.16). The interaction of methionine feeding treatment and sucrose antennal treatment was also not significant (*Χ_30_* ^2^ = 24.94, *p* = 0.73).

### Total consumption and single dose mortality

Honey bee foragers were fed a single dose of Se or sulfur as selenate, selenomethionine, or methionine plus sucrose at 5 concentrations, and then mortality was scored for 5 days. Treatments were compared to bees fed 1 M sucrose as the control. There was no significant difference in total consumption of selenate (ANOVA, *F_5,232_* = 0.79, *p* = 0.56), selenomethionine (*F_5,108_* = 1.26, *p* = 0.29) or methionine (*F_5,129_* = 2.19, *p* = 0.06) at the 5 concentrations. Bees ingested an overall average of 21.94±0.47 µl of selenate in 1 M sucrose (N = 18–21), 21.83±0.97 µl of selenomethionine in 1 M sucrose (N = 18–20), and 20.51±0.63 µl of methionine in 1 M sucrose (N = 21–24) across all concentrations.

**Figure 7:**
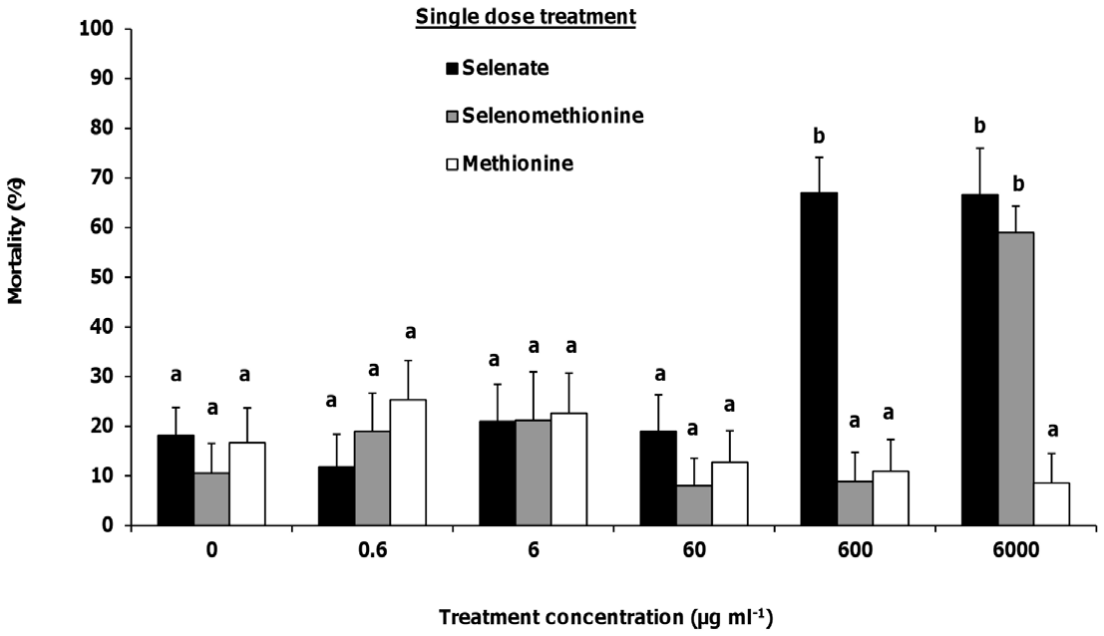
Honey bee forager mortality from a single dose of selenium. Percentages of honey bee mortality after a single dosage of selenate (N = 20–22), selenomethionine (N = 17–20) or methionine (N = 21–24) in 1 M sucrose at 6 concentrations. Control bees received 0 µg ml^−1^, or 1 M sucrose only. Mortality was recorded for 5 subsequent days. Final percent mortality is shown. Letters above the means indicate statistically significant differences between groups (α = 0.05) using the Mann-Whitney U test. Values are means ± standard error (SE).

Single dosage with selenate significantly increased final percent mortality in honey bee foragers at the 600 µg ml^−1^ (Kruskal-Wallis, *Χ_1_* ^2^ = 29.83, *p*<0.0001) and 6000 µg ml^−1^ (*Χ_1_* ^2^ = 37.31, *p*<0.0001) treatment levels compared to 1 M sucrose (Figure 7). Mortality reached as high as 67% at the 6000 µg ml^−1^ selenate concentration. Selenomethionine consumption also had a significant effect on mortality (Figure 7), and increased mortality to 59% at the highest concentration (*Χ_1_* ^2^ = 24.22, *p*<0.0001). Methionine consumption had no significant effect on mortality at all concentrations (Figure 7). Overall mortality across all methionine concentrations ranged from 9 to 23%.

### Chronic dose mortality

**Figure 8:**
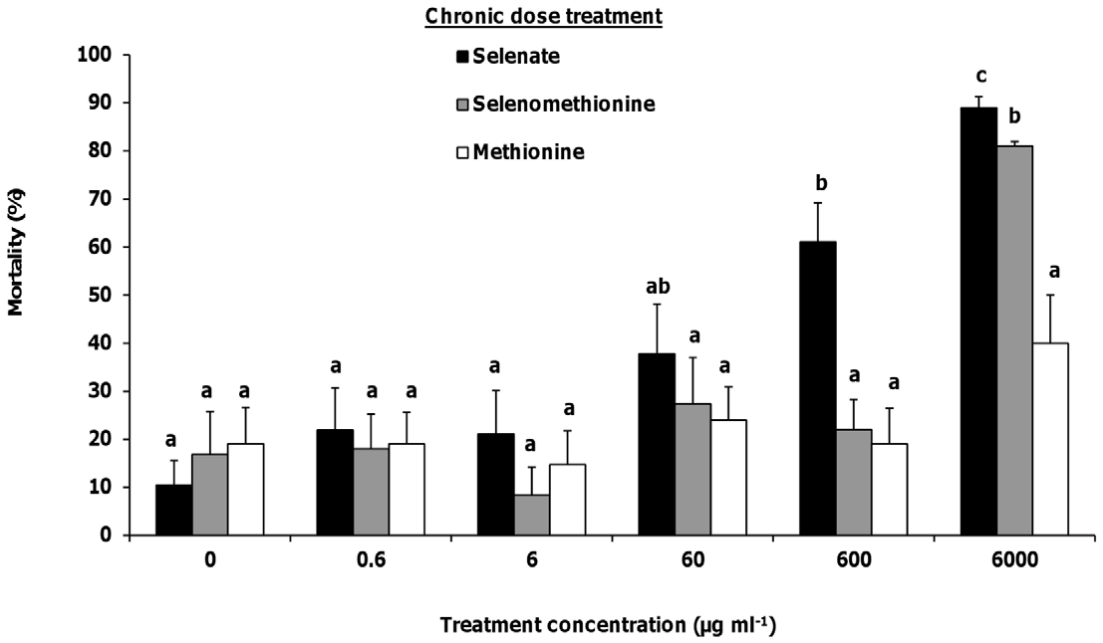
Honey bee forager mortality from chronic doses of selenium. Percentages of honey bee mortality after chronic dosage of selenate (N = 18–21), selenomethionine (N = 19–20) or methionine (N = 19–20) in 1 M sucrose at 6 concentrations. Control bees received 0 µg ml^−1^, or 1 M sucrose only. Bees were fed 20 µl of each treatment for 6 days. Mortality was recorded for 5 subsequent days after the first dosage. Final percent mortality is shown. Letters above the means indicate statistically significant differences between groups (α = 0.05) using the Mann-Whitney U test. Values are means ± standard error (SE).

Honey bee foragers were fed Se or sulfur as selenate, selenomethionine, or methionine plus sucrose at 5 concentrations for 5 days, and then mortality was scored on each day. Treatments were compared to bees fed 1 M sucrose as the control. Chronic dosing with selenate significantly increased mortality (Figure 8) at the 60 µg ml^−1^ (*Χ_1_* ^2^ = 5.40, *p*<0.02), 600 µg ml^−1^ (*Χ_1_* ^2^ = 17.81, *p*<0.0001) and 6000 µg ml^−1^ (*Χ_1_* ^2^ = 32.84, *p*<0.0001) concentrations compared with bees fed 1 M sucrose. Selenate consumption for 5 days increased mortality to as high as 89% in the 6000 µg ml^−1^ concentration. Similar to single dose mortality, chronic doses of selenomethionine increased mortality only at the highest concentration (*Χ_1_* ^2^ = 24.70, *p*<0.0001; Figure 8), although more bees died with a chronic dose (81%) compared to the single dose (59%). Chronic dosing with methionine at all concentrations did not have a significant effect on mortality (*Χ_1_* ^2^<3.19, *p*>0.07), although mortality was higher for chronic dosing compared to single dosing in the 6000 µg ml^−1^ treatment group (13% vs. 40%).

## Discussion

Our first objective examined whether the presence of Se affected the gustatory behaviors of honey bees via two different chemosensory organs (antenna or proboscis). Honey bee sensillae used to taste sugars and salts have been found on mouthparts associated with the proboscis [38]–[40]. Taste sensilla on the antennae respond to sugars [41] and salt solutions [42]. Se deters feeding in certain insect [30], [43], [44] and mammalian [45], [46] herbivores, and may reduce feeding behaviors such as PER in honey bees. However, some insects cannot detect Se and will ingest it in laboratory feeding studies [47], [34]. In our study, the presence of selenate in sucrose did not reduce the responses of honey bees to stimulation of the antennae or proboscis. However, antennal stimulation with selenomethionine significantly reduced PER at 600 µg ml^−1^ and higher, indicating that there was some decrease in response. Antennal stimulation with methionine also reduced PER at higher concentrations, suggesting that deterrence may be due to the methionine portion of the selenomethionine molecule. Methionine causes behavioral deterrence in the leaf-chewing herbivores *Spodoptera litura* F. (Lepidoptera: Noctuidae) [48], *Grammia geneura* Strecker (Lepidoptera: Arctiidae) [49] and *Mamestra brassicae* L. (Lepidoptera: Noctuidae) [50] under experimental conditions. Selenomethionine and methionine may interfere with the honey bee's perception of the sucrose reward when antennae are stimulated, thus reducing PER. In a study by de Brito Sanchez et al. [42], antennal stimulation with solutions containing sucrose and the bitter substance quinine inhibited PER and reduced electrophysiological responses to sucrose in the honey bee. Alternatively, honey bees may respond less to the amino acid depending on the amount of amino acid already in their hemolymph prior to capture [51]. Honey bees that have recently fed on a protein rich plant source may be less responsive to it in subsequent feeding stimulations.

In the proboscis response assays, the bees could choose to drink a small droplet of Se or sulfur containing sucrose solution. There was no significant difference between consumption of the 1 M sucrose alone (control) and selenomethionine or methionine in 1 M sucrose treatment droplets, indicating that the decrease in response was mediated by the antennae and not the proboscis. Honey bee foragers prefer to feed upon sugar solutions containing certain amino acids [51], [52], [53]. Methionine is an essential amino acid for honey bee development [54], although higher concentrations in nectar may act as a deterrent. In our study, deterrence was specific to antennal stimulation, suggesting that receptors detecting either methionine or selenomethionine may not be present on the proboscis.

Our second objective examined the effects of Se ingestion on the sucrose responsiveness of honey bees. Foraging honey bees evaluate floral resources based on the sugar concentrations in nectar, and adjust their foraging and recruitment behaviors accordingly [55]. The sucrose response threshold is an important benchmark for bees to recruit to a floral resource. In our study, the sucrose response threshold, or the point when the probability of responding to sucrose was significantly greater than water, was not significantly altered by feeding honey bees with Se compounds or methionine prior to testing for sucrose responsiveness. However, selenate did significantly reduce the overall responsiveness of the foragers to sucrose as fewer bees fed selenate responded to any sucrose concentration compared to bees fed 1 M sucrose alone. Selenate may lower the honey bee's overall level of responsiveness and arousal, reducing its ability to evaluate relevant stimuli such as a rich floral resource. Honey bees fed toxins such as ethanol [56], the pesticides fipronil [57], or thiamethoxam [58] showed reduced responsiveness to sucrose. If honey bee foragers ingest nectar containing selenate, foraging behaviors may be altered and bees may be less responsive to floral resources.

Our third objective examined the lethal effects of Se ingestion in honey bee foragers when applied at single or chronic dosages. Se as a micronutrient is essential for survival, but higher concentrations can be toxic to insects [31]. Se ingestion increased mortality and development time in *Cotesia marginiventris* Cresson (Hymenoptera: Braconidae) [59], *Heliothis virescens* F. (Lepidoptera: Noctuidae) [60], *P. maculiventris* [61] and *S. exigua* [30], [33], [34]. In our study, selenate was more toxic than selenomethionine or methionine when fed to honey bee foragers as either a single or chronic dose. Selenomethionine was toxic only at the highest dosage. In other insect plant-feeders, selenomethionine was as toxic as selenate in *S. exigua* [33], but more toxic than selenate in *H. virescens* [60]. In the detritivore *Megaselia scalaris* Loew (Diptera: Phoridae), selenomethionine was more toxic than selenate [47]. In insects fed various forms of Se, selenocompounds concentrated in the hindgut of the Se-tolerant *Plutella xylostella* L. (Lepidoptera: Plutellidae) [62], whereas Se concentrated in the Malpighian tubules of the Se-intolerant *Tenebrio molitor* L. (Coleoptera: Tenebrionidae) [63], suggesting these are the sites of sequestration and detoxification. Se detoxification in tolerant insects has been attributed to their ability to sequester Se as methylated forms of selenocompounds [62], which can prevent their misincorporation into proteins. In addition, trimethylselenonium-like species were found in the parasitoid *C. marginiventris*, suggesting they may detoxify the selenium accumulated from contaminated hosts by using methylation and volatilization [59]. Honey bees may employ similar mechanisms of detoxification by methylating or even volatilizing the Se.

Bees chronically fed 60 µg ml^−1^ selenate and higher experienced a significant decrease in survival. Greenhouse studies irrigating *Brassica juncea* (Indian mustard) [64] and *Raphanus sativus* (radish, unpublished data) with selenate treatment levels comparable to contaminated water in the western San Joaquin Valley of California revealed flowers accumulated up to 60 µg Se ml^−1^ Se in the nectar of both plant species. In the field, plants growing in soils containing 5 to 10 mg Se kg^−1^ accumulated approximately 1800 mg Se kg^−1^ dw in their flowers [65], and insect floral visitors to hyperaccumulator and non-accumulator plants contained an average of 44 and 10 µg Se g^−1^ dw respectively [23]. For the hyperaccumulator plant *Stanleya pinnata*, flowers accumulated 2323 mg Se kg^−1^ dw, with nectar containing 244 µl Se ml^−1^ fw [19]. Pollen collected by bees from New England aster growing in fly ash from coal-burning electrical power plants contained 14 mg Se kg^−1^ [16]. Floral visitors on Se-accumulating plants contained up to 75 µg Se g^−1^ dw [23], honey bees contained 14.8 µg Se g^−1^ dw and bumble bees contained 251 µg Se g^−1^ dw [19] when collected from seleniferous field sites in Colorado. Several greenhouse and field studies suggest there is the potential for honey bee foragers to acquire toxic levels of Se from certain species of plants growing in Se-contaminated areas.

Se in plant tissue or artificial diet has been shown to have negative effects on several insect species, yet we know very little about how soil-borne pollutants can impact pollinators. Insect herbivores fed plant tissues containing high levels of metals, metalloids (such as Se), or other accumulated elements have shown reduced development and survival [32], and several studies have shown some insect species cannot detect detrimental levels of Se [33], [34]. If nectar contains Se in the form of selenate, honey bees may not avoid these plants. If the foraging honey bees feed on nectar containing Se (particularly selenate), reductions in population numbers may occur due to direct toxicity. The older, foraging population of workers may be reduced, and younger workers may need to precociously forage to maintain the constant flow of resources into the colony. On the other hand, if the nectar contains selenomethionine, bees may detect and avoid these flowers. Additionally, our study has shown that fewer bees respond to sucrose when fed selenate. If a forager bee does survive the ingestion of selenate, she may be less responsive, forage and recruit less, and not properly evaluate valuable floral resources. Fewer responsive foragers may reduce the incoming floral resources needed to support coworkers and larvae. Taken together, effects on survival and foraging behaviors may significantly reduce the productivity and longevity of the colony. Our study is the first to examine the sublethal and lethal effects of a plant-accumulated pollutant on honey bee feeding preference, sucrose response threshold and mortality.

## Materials and Methods

### Compounds tested

Sodium selenate (henceforth, selenate, Na_2_SeO_4_, 98% purity), seleno-DL-methionine (henceforth, selenomethionine, C_5_H_11_NO_2_Se, 99% purity) and DL-methionine (henceforth, methionine, C_5_H_11_NO_2_S, 99% purity) were all purchased from Sigma-Aldrich (St. Louis, MO). These forms of Se were chosen for comparison to toxicity assays using *Spodoptera exigua* Hübner (Lepidoptera: Noctuidae) [33], [30], [34]. Compounds were prepared at 5 treatment levels so that each treatment contained the following concentrations of Se or sulfur: 0.6 µg ml^−1^, 6 µg ml^−1^, 60 µg ml^−1^, 600 µg ml^−1^, and 6000 µg ml^−1^. A control containing 0 µg ml^−1^ (1 M sucrose alone) was also included. In previous experiments, two non-hyperaccumulator plant species, *Brassica juncea* L. (Indian mustard) [64] and *Raphanus sativus* L. (radish) (unpublished data), accumulated up to 60 µg ml^−1^ total Se in the nectar when irrigated with selenate in the greenhouse. Therefore treatments included this concentration and two orders of magnitude higher and two lower. Stock solutions were prepared in 1 M sucrose solution (99.9% purity, Fisher Scientific, Pittsburg, PA). Sucrose solution alone and deionized water alone were used for control treatments.

### Animals

Tests were performed from June 2010 until January 2011 at the University of California – Riverside (UCR, Riverside, CA) using honey bee (*A. mellifera*) foragers collected at the entrance of a hive maintained at Agricultural Operations at UCR. The queen was not changed during the course of these experiments to minimize genetic variation. Bees were captured in small glass scintillation vials and chilled briefly at 4°C until immobile. Each individual was restrained in a harness comprised of a 3.8 cm long piece of drinking straw with a diameter of 7 mm. A thin strip of duct tape secured between the head and thorax permitted movement of the antennae and proboscis. Each bee was fed *ad libitum* with 1 M sucrose solution after harnessing. Bees were then left for 24 hours in a humid box at room temperature within the laboratory before use in experiments.

### Antennal response assays

Honey bee taste sensillae have been found on mouthparts associated with the proboscis [38], [39], [40], as well as on the antennae [41], therefore we tested the bee's response to stimulation of both. Honey bee foragers were tested with a range of Se concentrations to determine whether they would respond with PER to antennal stimulation with Se. Assays were based on methodology from de Brito Sanchez et al. [42], and delivered the test compound dissolved in 1 M sucrose to the antennae, eliciting PER. PER responses were scored as (+), proboscis extended upon antennae stimulation, or (−), proboscis retained after antennae stimulation. Bees that did not extend their proboscis even when their antennae were stimulated with sucrose were recorded as non-responsive. We determined the response thresholds for 2 Se compounds (selenate and selenomethionine) and 1 sulfur compound (methionine) dissolved in 1 M sucrose at 5 concentrations (0.6 µg ml^−1^, 6 µg ml^−1^, 60 µg ml^−1^, 600 µg ml^−1^, and 6000 µg ml^−1^). In addition, 1 M sucrose only and water only touched to the antennae served as controls. Bees were stimulated with solution contained within a Gilmont micrometer glass syringe (Gilmont Instruments, Barrington, IL). Immediately before the assay, each honey bee was tested for their motivation to feed by touching the antennae with a droplet of 1 M sucrose solution and observing the proboscis extension. Only bees extending their proboscis were chosen for subsequent trials. Antennal stimulation with water in between each treatment stimulation served as a control for sensitization, with an intertrial time of about 3 minutes.

### Proboscis response assays

Proboscis response assays were based on methods used in Wright et al. [40]. Each bee's antenna was stimulated with a 1 M sucrose droplet to elicit the PER [66], then each bee was fed 0.6 µl of the treatment solution. The proboscis stimulation treatment involved exposing the proboscis to selenate, selenomethionine, or methionine dissolved in 1 M sucrose solution in a 0.6 µl droplet administered with a Gilmont syringe. The small volume used to stimulate proboscises ensured that bees would not feed enough to reach satiation and become less responsive. Groups of bees were tested with either selenate, selenomethionine or methionine dissolved in 1 M sucrose at 5 treatment concentrations (0.6 µg ml^−1^, 6 µg ml^−1^, 60 µg ml^−1^, 600 µg ml^−1^, and 6000 µg ml^−1^ as Se or sulfur). Proboscis exposure to a droplet of 1 M sucrose or water acted as positive and negative controls, respectively. Bees were scored as (+), bee consumed entire 0.6 µl droplet, or (−), bee did not consume droplet.

### Sucrose response threshold assays

To examine the effects of the consumption of selenium on the responsiveness to sugars, bees were fed an acute dose of selenate, selenomethionine or methionine and then their sucrose response thresholds were determined. The sucrose response threshold assays were based on methods from Mustard et al. [56] and Page et al. [55]. Honey bees were captured and harnessed as described above. Twenty four hours later, bees were fed 20 µl solutions of 1 M sucrose containing 0 (control), 0.6 µg ml^−1^, 6 µg ml^−1^, 60 µg ml^−1^, 600 µg ml^−1^, and 6000 µg ml^−1^ of Se or sulfur in the forms of selenate, selenomethionine or methionine. Two hours after the bees had consumed the treatment, they were assayed for sucrose response threshold. Each bee's antennae were stimulated with sucrose solutions at increasing concentrations of 0.1%, 0.3%, 1%, 3%, 10% and 30%, interspersed with antennal stimulation with water. Water stimulations were interspersed between sucrose stimulations to serve as a control for increased sensitization or habituation on subsequent responses from repeated sucrose stimulation. After antennae were stimulated, proboscis extension (+) or retention (−) was recorded. Intertrial times were 3 minutes.

### Total consumption and single dose mortality assays

Bees were captured and harnessed as described above and fed 1 M sucrose only *ad libitum*. Twenty four hours later, bees were fed treatments of Se or sulfur as selenate, selenomethionine, or methionine dissolved in 1 M sucrose at 6 concentrations (0, 0.6, 6, 60, 600, and 6000 µg ml^−1^) for a total of 18 treatment groups. Bees were fed using a Gilmont syringe. The total volume consumed from each treatment was calculated. Bees remained harnessed for 5 days after the single dosage and mortality per day was scored in control and treated groups and has been presented as final mortality after 5 days. Surviving bees were fed 1 M sucrose *ad libitum* on each of the 5 subsequent days.

### Chronic dose mortality assays

Based on the average volume of treatment solution consumed in each treatment in the single dose assay, bees were fed 20 µl for each control and treated group on day 0, and were fed an additional 20 µl of treatment solution on each of the 5 subsequent days. Treatments consisted of selenate, selenomethionine, or methionine dissolved in 1 M sucrose at 6 concentrations (0, 0.6, 6, 60, 600, and 6000 µg ml^−1^ as Se or S) for a total of 18 treatment groups. Throughout the assay, bees were evaluated in control and treated groups for mortality per day.

### Statistical analysis

Antennal response, proboscis response, and sucrose threshold response probabilities were analyzed as a binary variable using repeated-measures logistic regression with each bee as a unit of replication. Data were analyzed using the GENMOD procedure in SAS (version 9.2, SAS Institute, Cary, NC) with *post hoc* multiple comparisons. Antennal and proboscis response compared PER probabilities in the 1 M sucrose control group to the treated groups unless otherwise noted. Sucrose response threshold assays compared response probabilities between the water trials and each sucrose concentration. Total consumption was analyzed for each treatment group using ANOVA (GLM procedure) and *post hoc* Tukey's HSD test. For mortality assays, as recommended in the EPA Ecological Effects Test Guidelines (OPPTS 850.3020), mortality was 20% or less in all control groups. Based on preliminary studies feeding harnessed foragers with 1 M sucrose, mortality increased above 20% by day 6, therefore we concluded the toxicity bioassays at day 5. Each honey bee represented a unit of replication. Pairwise comparisons were made of mortality in the 1 M sucrose (control) group to each treatment level and within each Se form. Se forms were not compared to each other. Mortality data was not normally distributed; therefore comparisons were made using the nonparametric Kruskal-Wallis test with *post hoc* separations using the Mann-Whitney *U* test (NPAIR1WAY procedure).

## Supporting Information

Table S1 **Honey bee sucrose response thresholds after selenium feeding treatments.**
(XLS)
